# Phylogenetic and genomic analyses of two new species of *Clavispora* (*Metschnikowiaceae*, *Saccharomycetales*) from Central China

**DOI:** 10.3389/fmicb.2022.1019599

**Published:** 2022-10-13

**Authors:** Chun-Yue Chai, Ying Li, Zhen-Li Yan, Feng-Li Hui

**Affiliations:** ^1^College of Life Science and Agricultural Engineering, Nanyang Normal University, Nanyang, China; ^2^Research Center of Henan Provincial Agricultural Biomass Resource Engineering and Technology, Nanyang Normal University, Nanyang, China; ^3^State Key Laboratory of Motor Vehicle Biofuel Technology, Henan Tianguan Enterprise Group Co., Ltd, Nanyang, China

**Keywords:** *Clavispora paralusitaniae* sp. nov., taxonomy, rotted wood-inhabiting yeast, genomic analyses, phylogeny, *Clavispora xylosa* sp. nov.

## Abstract

Species in the genus *Clavispora* have previously been reported primarily in the northeast and northwest regions of China; the species diversity of *Clavispora* in central China is not currently clear. In this study, phylogenetic inferences of *Clavispora* based on sequences of a single-locus (LSU D1/D2) and a two-locus (LSU D1/D2 and ITS) were conducted. Two new species isolated from rotting wood in central China, namely *Clavispora xylosa* sp. nov. and *Clavispora paralusitaniae* sp. nov., were delimited and proposed based on morphological and molecular evidence. *Cl. xylosa* was closely related to *C. thailandica* CBS 10610^T^, but with 11.5% divergence in the LSU D1/D2 domains and 11.5% divergence in the ITS regions. *Cl. paralusitaniae* was a sister to *Cl. lusitaniae* CBS 6936^T^ from which it differs with 4.7% divergence in the LSU D1/D2 domains and 5.4% divergence in the ITS regions. Description of *Cl. xylosa* sp. nov. and *Cl. paralusitaniae* sp. nov. was also supported by morphological comparisons and genomic analyses between the two new species and their closest relatives, *C. thailandica* CBS 10610^T^ and *Cl. lusitaniae* CBS 6936^T^. These results indicate a potentially great diversity of *Clavispora* spp. inhabiting rotting wood in central China, ripe for future discovery.

## Introduction

The genus *Clavispora* was established by Rodrigues de Miranda and is typified by *Clavispora lusitaniae*, the teleomorph of *Candida lusitaniae* (Rodrigues de Miranda, 1979; Lachance and Phaff, 2011). Later, *Lodderomyces opuntiae* was reclassified as *Cl. lusitaniae* because its ascospore is clavate in shape (Phaff et al., 1986). Yurkov et al. (2009), identified yeasts isolated from soils and proposed a new member of the genus *Clavispora*, *Clavispora reshetovae*, based on its morphology and phylogenetic placement. In addition, due to an inability to form sexual spores, some yeast species that belong to the *Clavispora* clade as revealed by phylogenetic analysis based on DNA sequences were previously placed in the anamorphic genus *Candida* (Lu et al., 2004; Jindamorakot et al., 2007; Nguyen et al., 2007; Rosa et al., 2007; James et al., 2009; Fell et al., 2011; Groenewald et al., 2011; Lachance et al., 2011; Nakase et al., 2011; Ribeiro et al., 2011; Limtong and Kaewwichian, 2013; Zhang et al., 2014). With the implementation of the “one fungus, one name” nomenclature, the relationships between *Candida* and *Clavispora* species began to be clarified (Daniel et al., 2014; Kurtzman et al., 2018). Daniel et al. (2014) provisionally reassigned 40 *Candida* species that are not related to the genus *Metschnikowia* to the genus *Clavispora*, although their monophyletic origin is doubtful. Recently, *Candida fructus* has been transferred to *Clavispora* as a new combination, and *Clavispora santaluciae* and *Candida xylosifermentans* have also been included as members of the *Clavispora* clade (Kurtzman et al., 2018; Kaewwichian et al., 2019; Drumonde-Neves et al., 2020). The genus *Clavispora* belongs to the family Metschnikowiaceae in the order Saccharomycetales, and is closely related to the genus *Metschnikowia* based on multigene phylogenetic analyses (Kurtzman and Robnett, 2013; Kurtzman et al., 2018). To date (December 2019), the YeastIP database (Weiss et al., 2013) lists 33 members of this clade, but the actual number of species assigned to the clade has reached 35 (Kaewwichian et al., 2019; Drumonde-Neves et al., 2020): five species of the genus *Clavispora* and 30 asexual species still assigned to the genus *Candida*.

The habitats of species belonging to the *Clavispora* clade are very diverse and they have been found in plants (Lu et al., 2004; Rosa et al., 2007; Groenewald et al., 2011; Lachance et al., 2011; Ribeiro et al., 2011; Limtong and Kaewwichian, 2013; Zhang et al., 2014; Drumonde-Neves et al., 2020), soil (Yurkov et al., 2009; Nakase et al., 2011; Limtong and Kaewwichian, 2013), insects (Nguyen et al., 2007; Lachance et al., 2011), insect frass (Jindamorakot et al., 2007; Lachance et al., 2011; Nakase et al., 2011), fish (Lachance et al., 2011), fresh water (Fell et al., 2011), moss (Lachance et al., 2011; Kaewwichian et al., 2019), and clinical samples (Lachance and Phaff, 2011). *Cl. lusitaniae* can be pathogenic to humans and is responsible for about 1% of invasive candidiasis cases, particularly in pediatric and hematology–oncology patients (Merz et al., 1992; Favel et al., 2003). Some species in this clade, such as *Candida akabanensis*, *C. xylosifermentans*, and *Cl opuntiae,* were found to ferment D-xylose, which is the second most abundant sugar in plant cell-wall carbohydrates (Phaff et al., 1986; Lachance et al., 2011; Valinhas et al., 2018; Kaewwichian et al., 2019). The ability of yeasts to ferment D-xylose is of interest because such yeasts can be applied to bioethanol production from lignocellulosic biomass (Agbogbo and Wenger, 2007; Urbina et al., 2013). Therefore, *Clavispora* species are important not only due to their pathogenicity in humans, but also for their potential applications in food and biofuels.

Species in the *Clavispora* clade have a worldwide distribution, but most were originally identified in Asia (Lu et al., 2004; Jindamorakot et al., 2007; Lachance et al., 2011; Nakase et al., 2011; Limtong and Kaewwichian, 2013; Zhang et al., 2014; Kurtzman et al., 2018; Kaewwichian et al., 2019), Europe (Yurkov et al., 2009; Drumonde-Neves et al., 2020), North America (Nguyen et al., 2007; Fell et al., 2011; Lachance et al., 2011), or South America (Rosa et al., 2007; Groenewald et al., 2011; Ribeiro et al., 2011). Over the last several years, this clade has received a great deal of attention in Asia, with two novel species found in Japan (Lachance et al., 2011; Nakase et al., 2011) and five novel species found in Thailand (Jindamorakot et al., 2007; Nakase et al., 2011; Limtong and Kaewwichian, 2013; Kaewwichian et al., 2019). In contrast, little is known about *Clavispora* spp. in China. To date, only two species of the *Clavispora* clade have been reported in China: *Candida asparagi* and *Candida pruni* (Lu et al., 2004; Zhang et al., 2014).

The aim of the current study is to explore the species diversity of *Clavispora* in central China, and more importantly, to construct a more natural taxonomic system of *Clavispora*, based on phylogenetic analyses. In addition, genomic data that can be analyzed with tools to determine species delineation is explored.

## Materials and methods

### Sample collection and yeast isolation

Rotting wood samples were collected in two areas of Henan Province in central China, in the Funiu Mountain Nature Reserve (32°45′N, 113°30′E) and in the Baotianman Nature Reserve (33°27′N, 111°48′E). The predominant vegetation was characterized in those areas as a warm-temperate to subtropical forest biome. The climate was warm-temperate, with an annual precipitation volume of 885.6 mm and an average temperature of 15.2°C. Yeasts were isolated from rotting wood samples as previously described (Kaewwichian et al., 2019; Lv et al., 2020). Briefly, each wood sample (1 g) was added to 20 ml sterile D-xylose medium (yeast nitrogen base 0.67%, D-xylose 0.5%, and chloramphenicol 0.02%, pH 5.0 ± 0.2) in a 150 ml Erlenmeyer flask and then cultured at 25°C for 3–10 days on a rotary shaker. Subsequently, 0.1 ml aliquots of the enrichment culture and appropriate decimal dilutions were spread on D-xylose agar plates and then incubated at 25°C for 3–4 days. Representative colonies were selected and the yeasts were isolated through repeated plating on D-xylose agar, and then stored on yeast extract-malt extract (YM; 1% glucose, 0.5% peptone, 0.3% yeast extract, and 0.3% malt extract; pH 5.0 ± 0.2) agar slants at 4°C or in 15% glycerol at −80°C. Strains of the two new species described in this paper are listed in Table 1.

**Table 1:** Novel yeast strains isolated from rotting wood.

Strain	Source	Location
*Clavispora xylosa*		
NYNU 174173^T^	Rotting wood	Baotianman Nature Reserve, Henan, China
NYNU 168193	Rotting wood	Funiu Mountain Nature Reserve, Henan, China
*Clavispora paralusitaniae*		
NYNU 167235	Rotting wood	Funiu Mountain Nature Reserve, Henan, China
NYNU 168424	Rotting wood	Baotianman Nature Reserve, Henan, China
NYNU 161120^T^	Rotting wood	Funiu Mountain Nature Reserve, Henan, China

### Phenotypic characterization

The morphological, physiological and biochemical characteristics of the two new species were determined using standard methods (Kurtzman et al., 2011). Carbon and nitrogen assimilation tests were performed using liquid media, and growth was observed for up to 4 weeks. Carbon fermentation was tested in a YP base media (1% yeast extract and 2% peptone, pH 5.0 ± 0.2), and Durham tubes were used to visualize carbon dioxide production. Growth was assessed at a range of temperatures (30, 35, 37, and 40°C) by streaking cells onto yeast extract peptone glucose (YPD) agar (2% glucose, 2% peptone, 1% yeast extract, and 2% agar) plates and incubating them for 2 weeks. Formation of true hyphae and pseudohyphae were investigated using the Dalmau plate method on both cornmeal (CM) and 5% malt extract (ME) agar plates. The beginning of the sexual stage was determined by incubating single or mixed cultures of each of the two strains on CM agar, 5% ME agar, potato-dextrose agar (PDA; 20% potato infusion, 2% glucose, 2% agar), and yeast carbon base plus 0.01% ammonium sulfate (YCBAS) agar at 15°C or 25°C for 6 weeks (Yurkov et al., 2009; Lachance and Phaff, 2011; Drumonde-Neves et al., 2020). Photomicrographs were taken using a Leica DM 2500 microscope (Leica Microsystems GmbH, Wetzlar, Germany) with a Leica DFC295 digital microscope color camera using bright field, phase contrast, and differential interference contrast (DIC) optics. Novel taxonomic descriptions and proposed names were deposited in MycoBank (http://www.mycobank.org; 12 September 2022).

### DNA extraction, amplification, and sequencing

Genomic DNA was extracted from the yeasts using an Ezup Column Yeast Genomic DNA Purification Kit (Sangon Biotech, Shanghai, China) following the manufacturer’s instructions. The internal transcribed spacer (ITS) regions and the D1/D2 domains of the large subunit (LSU) rRNA gene were amplified using the primer pairs ITS1/ITS4 (White et al., 1990) and NL1/NL4 (Kurtzman and Robnett, 1998), respectively. The PCR program was as follows: initial denaturation at 95°C for 3 min; 35 cycles of 94°C for 40 s, 56°C for 45 s, and 72°C for 1 min; and a final extension of 72°C for 10 min (Shi et al., 2021). PCR products were cleaned and sequenced by Sangon Biotech Inc.

### Phylogenetic analysis

Phylogenetic analyses were conducted using the novel sequences generated from the five strains isolated in this study and all reference sequences of related strains available in GenBank (Table 2). Alignments for the LSU D1/D2 and ITS loci were conducted using MAFFT v7.110 with default settings (Katoh and Standley, 2013) and manually corrected where necessary. To establish the identity of the isolates at the species level, phylogenetic analyses were conducted first with each locus individually, then using two loci (ITS and LSU D1/D2) together, that are widely used molecular “barcode” regions for fungi, with LSU D1/D2 particularly suitable for yeasts. Phylogenetic trees were constructed in MEGA11 software (Tamura et al., 2021) for each of the datasets using the maximum likelihood (ML) and neighbor-joining (NJ) methods. *Saccharomyces cerevisiae* CBS 1171^T^ was used as the outgroup (Kurtzman et al., 2018; Kaewwichian et al., 2019).

**Table 2:** Sequences used in molecular phylogenetic analysis.

Species name	Strain no.	Sample	Locality	GenBank accession no.	
				ITS	LSU D1/D2
*Candida akabanensis*	CBS 5039^T^	Exudate	Japan	NR_155003	EU100744
*C. asparagi*	CBS 9770^T^	Fruit	China	NR_155004	NG_055316
*C. berkhoutiae*	CBS 11722^T^	Frass	Thailand	KY101957	DQ400372
*C. blattae*	CBS 9871^T^	Insect	USA	NR_111407	NG_055375
*C. carvajalis*	CBS 11361^T^	Rotten wood	Ecuador	KY102022	AM946644
*C. dosseyi*	CBS 10313^T^	Insect	USA	NR_111406	NG_055374
*C. ezoensis*	CBS 11753^T^	Soil	Japan	KY102082	AB462347
*C. flosculorum*	CBS 10566^T^	Flower	Brazil	NR_137682	EF137918
*C. intermedia*	CBS 572^T^	Feces	Africa	NR_111248	NG_055404
*C. middelhoveniana*	CBS 12306^T^	Sugarcane	Brazil	NR_155005	NG_060817
*C. oregonensis*	CBS 5036^T^	Frass	USA	NR_155006	NG_055406
*C. phyllophila*	CBS 12671^T^	Phylloplane	Thailand	/	NG_055303
*C. pruni*	CBS 12814^T^	Plums	China	NR_159744	NG_064321
*C. pseudoflosculorum*	CBS 8584^T^	Flower	Guyana	NR_165968/	NG_055379
*C. pseudointermedia*	CBS 6918^T^	Fish paste	Japan	NR_155007	NG_055407
*C. sharkensis*	CBS 11368^T^	Fresh water	USA	MK503789	NG_055377
*C. thailandica*	CBS 10610^T^	Frass	Thailand	NR_159560	NG_064310
*C. tsuchiyae*	CBS 7195^T^	Moss	Japan	NR_155010	NG_055413
*C. vitiphila*	CBS 12672^T^	Phylloplane	Thailand	AB736148	NG_055304
*C. xylosifermentans*	CBS11573^T^	Moss	Thailand	LC440109	AB525240
*Clavispora fructus*	CBS 6380^T^	Banana	Japan	NR_111292	NG_055405
*Cl. lusitaniae*	CBS 6936^T^	Peel juice	Israel	NR_130677	NG_055408
*Cl. opuntiae*	CBS 7068^T^	Prickly pear	Australia	NR_155011	NG_055409
***Cl. paralusitaniae***	**NYNU 167235**	**Rotting wood**	**China**	**OP019831**	**OP019826**
***Cl. paralusitaniae***	**NYNU 168424**	**Rotting wood**	**China**	**OP019833**	**OP019830**
***Cl. paralusitaniae***	**NYNU 161120** ^**T**^	**Rotting wood**	**China**	**MF136066**	**MF136063**
*Cl. reshetovae*	CBS 11556^T^	Soil	Germany	NR_137723	NG_055376
*Cl. santaluciae*	CBS 16465^T^	Grapes	Portugal	MN967319	MN970158
***Cl. xylosa***	**NYNU 174173** ^**T**^	**Rotting wood**	**China**	**MG255724**	**MG255710**
***Cl. xylosa***	**NYNU 168193**	**Rotting wood**	**China**	**OP019828**	**OP019827**
*Metschnikowia bicuspidata*	CBS 5575^T^	Fresh water	USA	KY104192	U44822
*M. reukaufii*	CBS 5834^T^	Flower	Canada	NR_111252	U44825
*Saccharomyces cerevisiae*	CBS 1171^T^	/	/	NR_111007	AY048154

Entries in bold are newly generated for this study.

CBS, CBS-KNAW collections, Westerdijk Fungal Biodiversity Institute, Utrecht, Netherlands. NYNU, Microbiology Lab, Nanyang Normal University, Henan, China. ^T^type strain.

Analysis with the ML method was performed using the best-fit substitution model GTR+ I + G (Nei and Kumar, 2000), whereas analysis with the NJ method was performed using the Kimura 2-parameter model (Saitou and Nei, 1987). The confidence levels of the clades were estimated from 1,000 bootstrap replicates (Felsenstein, 1985).

### Genome sequencing and assembly

The genomes of the type strains of *C. thailandica* and *Cl. lusitaniae* are publicly available, whereas the genomes of the novel strains NYNU 174173^T^ and NYNU 161120^T^ were sequenced for this study. The genomic DNA of each strain was isolated using a phenol: chloroform extraction protocol (Schwartz and Sherlock, 2016). The genomic DNA of the strains were sequenced using long-read PacBio platforms with single-molecule real-time (SMRT) technology. Sequencing libraries were constructed using TruSeq DNA PCR Free (350) kits (Illumina) and run on an Illumina NovaSeq instrument at Beijing BerryGenomics Co., Ltd. (Beijing, China).

To generate whole genome assemblies, paired-end Illumina reads were processed with the meta-assembler pipeline iWGS v1.1 (Zhou et al., 2016). Briefly, this pipeline performed quality-based read trimming followed by k-mer length optimization, then used a range of state-of-the-art assemblers to generate several genome assemblies for each strain. The quality of each assembly was assessed using QUAST v3.1 (Gurevich et al., 2013), and the best assembly for each species was chosen based on N50 statistics and genome size as calculated with Quast v5.0.2 (Gurevich et al., 2013).

### Genomic analyses

The DNA G + C content was calculated and protein-coding open reading frames (ORFs) were predicted using Glimmer v3.02 (Delcher et al., 2007). We used RNAmmer v1.2 (Lagesen et al., 2007) and tRNAscan-SE v1.3.1 (Lowe and Eddy, 1997) to predict rRNAs and tRNAs, respectively. The average nucleotide identity (ANI) values were calculated between strains NYNU 174173^T^ and NYNU 161120^T^ genomes and those of the most closely related species using a web-based calculator (Yoon et al., 2017)^1^ and FastANI (Jain et al., 2018). To calculate the distances between genomes, digital DNA–DNA homology (dDDH) values were estimated using the Genome-to-Genome Distance Calculator 2.1.^2^ The dDDH values presented here were calculated using Formula 2 (I_XY_: = sum of identical base pairs over all HSPs), which estimates values based on the identities of high-scoring segment pairs (Meier-Kolthoff et al., 2013).

## Results

### Phylogenetic analysis

**Figure 1:**
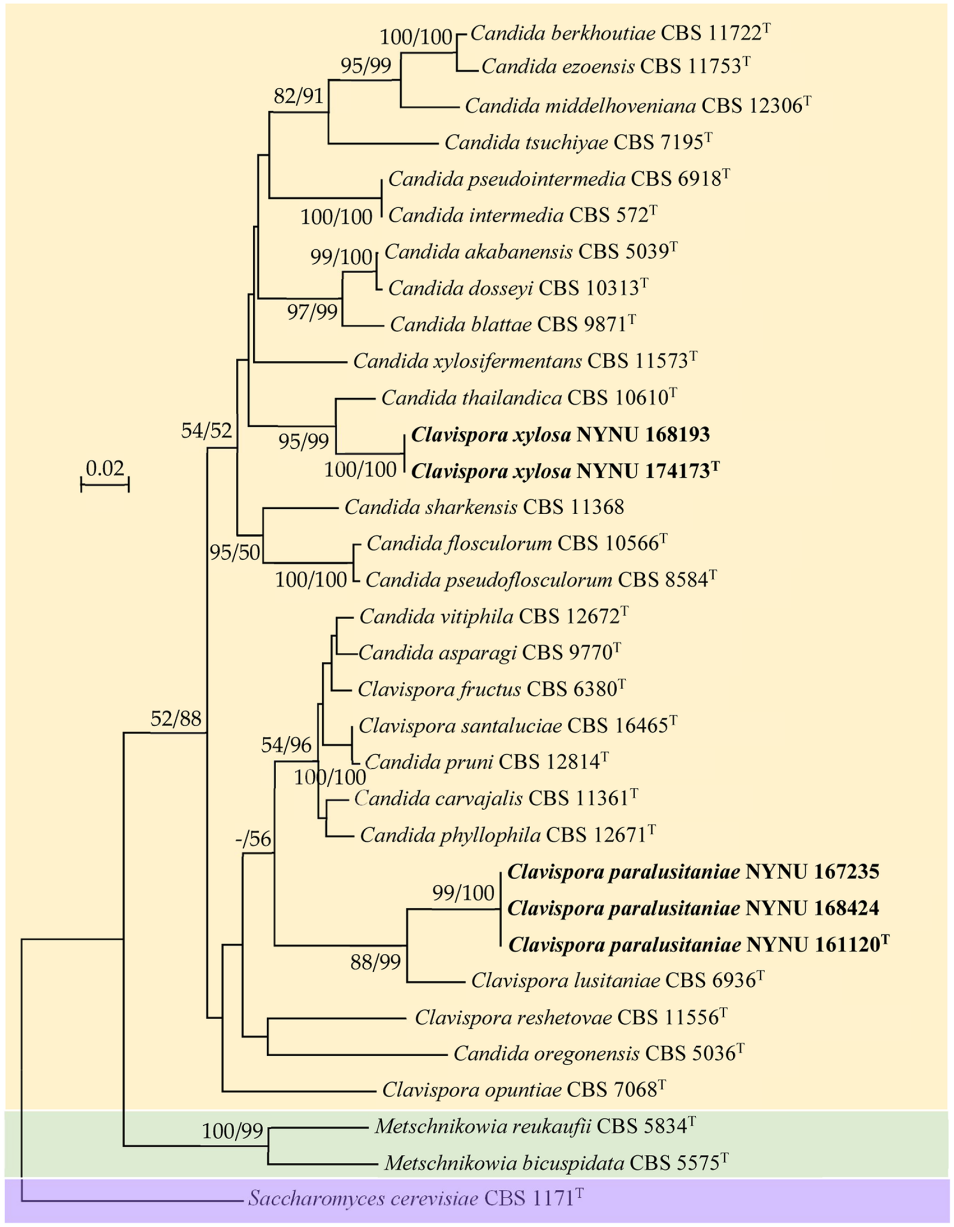
Maximum likelihood (ML) phylogram of *Clavispora* species based on the D1/D2 domains of the LSU rRNA gene. *Saccharomyces cerevisiae* CBS 1171^T^ was used as the outgroup. ML and neighbor-joining (NJ) bootstrap support values above 50% are shown at the nodes (ML/NJ). The novel strains described in this study are shown in bold.

**Figure 2:**
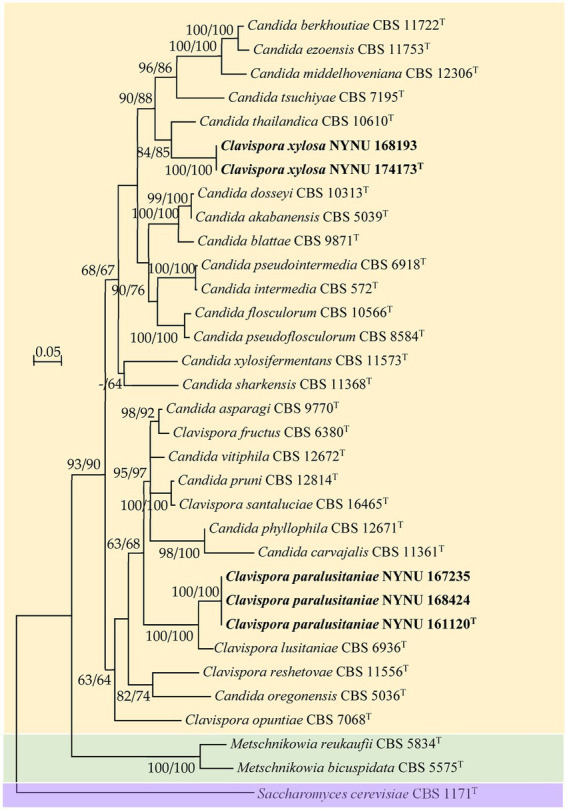
Maximum likelihood (ML) phylogram of *Clavispora* species based on the combined ITS + LSU D1/D2 sequence data. *Saccharomyces cerevisiae* CBS 1171^T^ was used as the outgroup. ML and neighbor-joining (NJ) bootstrap support values above 50% are shown at the nodes (ML/NJ). The novel strains described in this study are shown in bold.

Among the yeasts isolated from rotting wood samples collected in Henan Province, central China, five strains that could not be identified as known yeast species based on the rDNA sequences were selected for further taxonomic characterization. To establish the taxonomic position of the novel strains, phylogenetic analysis was carried out with the LSU D1/D2 sequences of the five novel strains and type strains of members of the *Clavispora* clade. The resulting phylogenetic trees showed that the five strains represented two new members of the *Clavispora* clade and could be classified into two taxa (Figures 1, 2). Three strains (NUNU 167235, NUNU 168424, and NYNU 161120) in group NYNU 161120^T^ had identical sequences in both the D1/D2 and ITS regions, which indicated that they are conspecific. Two other strains (NUNU 168193 and NYNU 174173) in group NYNU 174173^T^ had identical sequences in both the D1/D2 and ITS regions, which indicated that they are conspecific. The two new members of the *Clavispora* clade clearly distinguishable from those of other species, whether undescribed or previously characterized.

Strain NYNU 174173^T^ formed a well-delineated lineage with *C. thailandica* with a high bootstrap value (95% ML; 99% NJ; Figure 1) in the *Candida intermedia* subclade. Strain NYNU 174173^T^ differed by 11.5% divergence (24 substitutions and four gaps) from its close relative *C. thailandica* CBS 10610^T^ in the LSU D1/D2 domains and differed by 11.5% divergence (30 substitutions and 11 gaps) in the ITS regions. In accordance with the guidelines for yeast identification based on nucleotide divergences, yeast strains with 1% or more substitution in the D1/D2 domain or 1–2% nucleotide differences in the ITS region usually represent separate species (Kurtzman and Robnett, 1998; Scorzetti et al., 2002). The differences in both the LSU D1/D2 and ITS sequences were significant enough for this strain to be considered a novel species. The close relationship between strain NYNU 174173^T^ and *C. thailandica* CBS 10610^T^ was further confirmed by the combined ITS and LSU D1/D2 dataset (84% ML; 85% NJ; Figure 2).

Strain NYNU 161120^T^ clustered with *Cl. lusitaniae* with strong bootstrap support (88% ML; 99% NJ; Figure 1) in the *Cl. lusitaniae* subclade. Its nearest phylogenetic neighbor was *Cl. lusitaniae* CBS 6936^T^, from which strain NYNU 161120^T^ differed by 4.7% divergence (14 substitutions and two gaps) in the LSU D1/D2 domains and 5.4% divergence (24 substitutions and four gaps) in the ITS regions. According to the criteria mentioned above, the relatively low sequence similarity indicated that strain NYNU 161120^T^ and *Cl. lusitaniae* CBS 6936^T^ were distinct *Clavispora* species. The phylogenetic relationships between strain NYNU 161120^T^ and the previously described *Clavispora* species were further confirmed by the phylogenetic tree built from the combined ITS and LSU D1/D2 dataset (100% ML; 100% NJ; Figure 2).

### Phenotypic characterization

**Figure 3:**
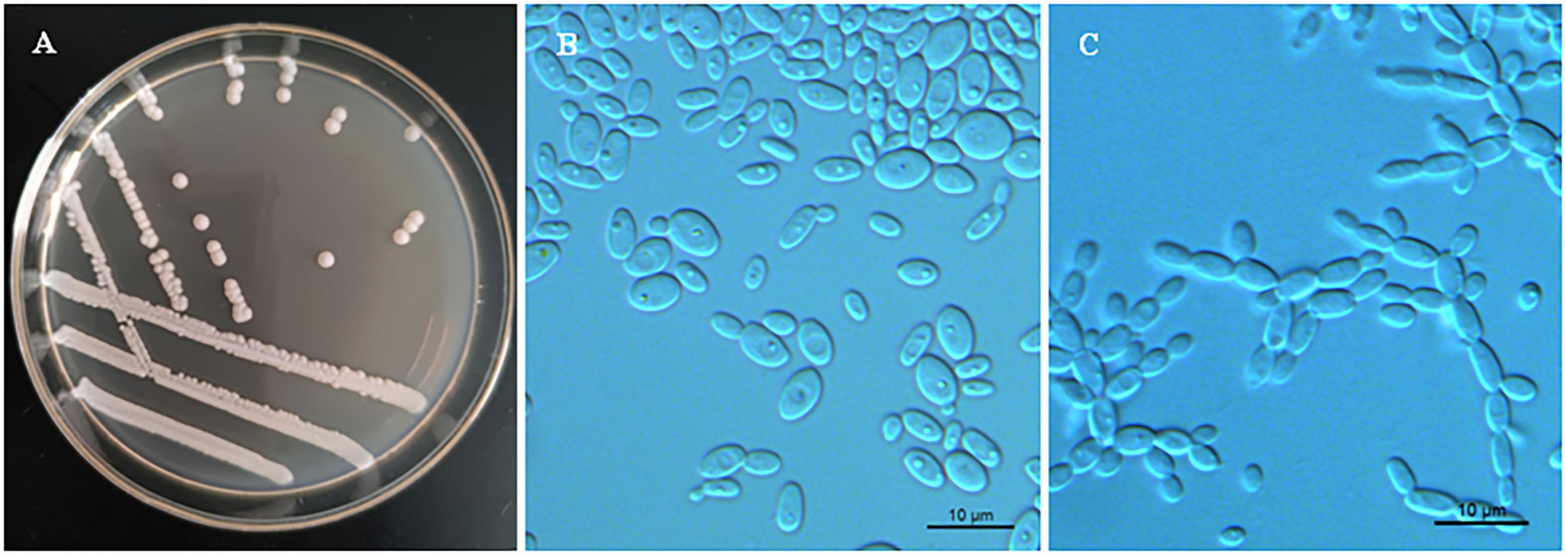
Morphological characteristics of *Clavispora xylosa* sp. nov. (CICC 33277, holotype). **(A)** Colony morphology on YM agar after growth for 3 days at 25°C. **(B)** Budding cells after growth for 3 d in YM broth at 25°C. **(C)** Pseudohyphae with blastoconidia on CM agar after growth for 7 days at 25°C. Scale bars = 10 μm.

**Figure 4:**
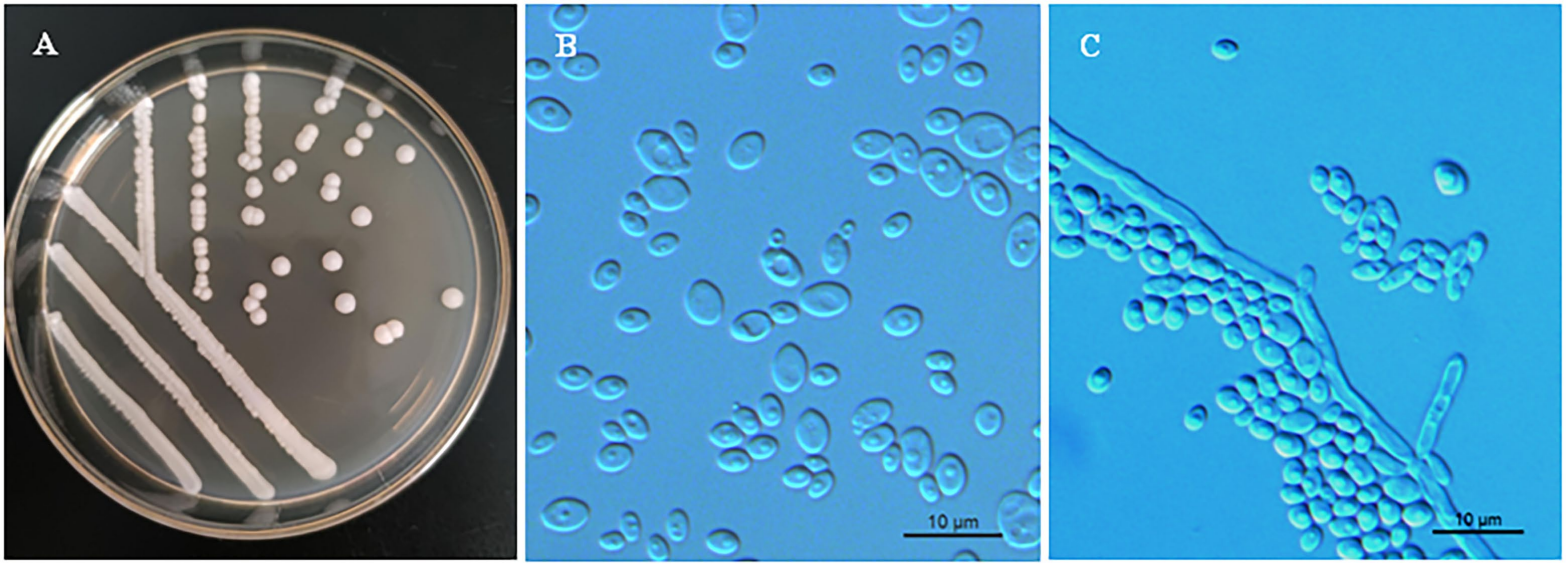
Morphological characteristics of *Clavispora paralusitaniae* sp. nov. (CICC 33276, holotype). **(A)** Colony morphology on YM agar after growth for 3 days at 25°C. **(B)** Budding cells after growth for 3 days in YM broth at 25°C. **(C)** Pseudohyphae with blastoconidia on CM agar after growth for 7 days at 25°C. Scale bars = 10 μm.

Phenotypic characterization was carried out for strains NYNU 174173^T^ and NYNU 161120^T^ using standard methods (Kurtzman et al., 2011). The yeasts shared similar phenotypic characteristics with other species in the *Clavispora* clade. Colonies were white to cream-colored, buttery, convex, and had an entire margin (Figure 3A, 4A). Cells were ovoid to elongate, proliferated by multilateral budding (Figure 3B, 4B), and formed pseudohyphae but not hyphae (Figure 3C, 4C). They were fermentative and could not assimilate nitrate as a nitrogen source. Their growth in vitamin-free medium was inconsistent with previous descriptions of the *Clavispora* clade (Jindamorakot et al., 2007; Yurkov et al., 2009; Lachance and Phaff, 2011), but studies of other closely related species demonstrated that this trait must be considered variable in this clade. Neither conjugation nor ascospores were observed in single or mixed cultures on sporulation media, suggesting that these strains represent anamorphs of the genus *Clavispora*.

Phenotypic characteristics that differed between strains NYNU 174173^T^ and NYNU 161120^T^ and closely related species in the *Clavispora* clade are shown in Table 3. Strain NYNU 174173^T^ could be differentiated from the most closely related known species, *C*. *thailandica* CBS 10610^T^ (Jindamorakot et al., 2007), based on fermentation of D-xylose; growth at 35°C; ability to assimilate L-arabinose and L-rhamnose; inability to assimilate D-arabinose, 2-keto-D-gluconate, DL-lactate, or cadaverine; and the inability to grow in 0.01% cycloheximide. Strain NYNU 161120^T^ could be morphologically differentiated from its nearest phylogenetic neighbor, *Cl*. *lusitaniae* CBS 6936^T^ (Lachance and Phaff, 2011), by the inability to produce one or two clavate ascospores in a liberated ascus. Physiologically, strain NYNU 161120^T^ could be differentiated from *Cl*. *lusitaniae* CBS 6936^T^ based on the ability to assimilate inulin; the inability to assimilate ethanol; and the inability to grow in 10% NaCl with 5% glucose and in 0.01% cycloheximide (Table 3).

**Table 3:** Phenotypic characteristics that differed between the novel strains NYNU 174173^T^ and NYNU 161120^T^ and their closely related taxa.

Characteristics	NYNU 174173^T^	*C. thailandica* CBS 10610^T^*	NYNU 161120^T^	*Cl. lusitaniae* CBS 6936^T*^
Fermentation of D-xylose	w	−	−	n
Assimilation of				
L-Arabinose	+	−	−	−
D-Arabinose	−	+	−	v
L-Rhamnose	+	−	+	+
Inulin	−	−	w	−
2-Keto-D-giuconate	−	+	+	+
DL-Lactate	−	+	w	v
Ethanol	−	−	−	+
Cadaverine	−	+	+	+
Growth tests				
10%Nacl/5%glucose	−	−	−	+
0.01% Cycloheximide	−	+	−	+
Growth at 35°C	+	−	**+**	+

+, positive reaction; −, negative reaction; w, weakly positive; v, variable reaction; n, data not available. All data from this study, except^*^ which were obtained from the original description (Phaff et al., 1986; Jindamorakot et al., 2007).

### Genomic analyses

The genomes of strains NYNU 174173^T^ and NYNU 161120^T^ were assembled and compared with those of the closest related species, *C*. *thailandica* CBS 10610^T^ and *Cl. lusitaniae* CBS 6936^T^, respectively. The detailed characteristics of these genomes are shown in Table 4. The genome of strain NYNU 174173^T^ is 15,305,418 bp in size, consisting of 10 scaffolds with an N50 length of 2,408,949 bp, and maximum and minimum scaffold lengths of 3,517,412 and 56,211 bp, respectively. The genome of strain NYNU 161120^T^ is 12,617,314 bp in size, consisting of eight scaffolds with an N50 length of 1,920,963 bp, and maximum and minimum scaffold lengths of 2,542,591 and 780,161 bp, respectively. A total of 4,930 genes, 4,647 ORFs, and 283 RNAs are predicted in strain NYNU 174173^T^, and a total of 5,085 genes, 4,774 ORFs, and 311 rRNAs are predicted in strain NYNU 161120^T^. The genomic G + C contents are 50.87 and 44.51% for strains NYNU 174173^T^ and NYNU 161120^T^, respectively; this is much higher than the G + C content in *C. thailandica* CBS 10610 ^T^ and *Cl. lusitaniae* CBS 6936^T^ (Table 4).

**Table 4:** Genome properties for strains NYNU 174173^T^ and NYNU 161120^T^ and for the type strains of the most closely related species in the *Clavispora* clade.

Attributes	NYNU 174173^T^	*C*. *thailandica* CBS 10610^T*^	NYNU 161120^T^	*Cl. lusitaniae* CBS 6936^T*^
Genome size (Mb)	15,305,418	16,310,147	12,617,314	11,992,787
Number of scaffolds	10	8	8	53
Scaffold N50 (bp)	2,408,949	2,767,786	1,920,963	699,681
Maximum scaffold size (bp)	3,517,412	3,371,249	2,542,591	1,779,567
Minimum scaffold size (bp)	56,211	594,379	780,161	1,006
GC content (mol%, genome)	50.87%	45.96%	44.51%	44.46%
Number of genes	4,930	5,782	5,085	5,740
Number of ORFs Protein-coding genes	4,647	5,516	4,774	5,537
Number of RNAs	283	266	311	201

All data from this study, except^*^ which were obtained from GenBank (https://www.ncbi.nlm.nih.gov/genbank/).

In yeast systematics, genomic data can be analyzed with tools that calculate genome-wide genetic distances between a novel taxon and its closest relatives to determine species delineation (Libkind et al., 2011; Lachance and Lee, 2020; Cadež et al., 2021). For estimation of genetic distances between strains NYNU 174173^T^ and NYNU 161120^T^ and their closest relatives, we used two tools that are available as web interfaces: the ANI calculator and the Genome-to-Genome Distance Calculator. The ANI and DDH values between the strain NYNU 174173^T^ and *C. thailandica* CBS 10610^T^ were 77.78 and 30.70%, respectively (Table 5); the ANI and DDH values between the strain NYNU 161120^T^ and *Cl. lusitaniae* CBS 6936^T^ were 94.72 and 53.50%, respectively. All these percentages were lower than 95 and 70%, respectively, which are the defined cut-off limits for species delineation (Lachance and Lee, 2020; Cadež et al., 2021), and further support the inclusion of these two strains as novel taxa in the *Clavispora* clade.

**Table 5:** Matrix of *in silico* DNA–DNA hybridization (DDH) and Ortho average nucleotide identity (OrthoANI) percentages between the genomes of strains NYNU 174173^T^ and NYNU 161120^T^ and the type strains of the most closely related species in the *Clavispora* clade.

ANI *In silico* DDH	NYNU 174173^T^	*C. thailandica* CBS 10610^T^	NYNU 161120^T^	*Cl. lusitaniae* CBS 6936^T^
NYNU 174173^T^		77.78%	71.69%	71.42%
*C. thailandica* CBS 10610^T^	30.70%		71.68%	71.51%
NYNU 161120^T^	32.50%	30.70%		94.72%
*Cl. lusitaniae* CBS 6936^T^	28.80%	26.90%	53.50%	

### Taxonomy

*Clavispora xylosa* F.L. Hui & C.Y. Chai sp. nov. (Figure 3).

*Mycobank No.*: 845627.

*Type*. China, Henan Province, Baotianman Nature Reserve, rotting wood, 3 August, 2016, NYNU 174173 (holotype CICC 33277, culture ex-type CBS 15236).

*Etymology*. *Clavispora xylosa* (xy.lo’sa. N.L. masc. adj. *xylosa*, of or pertaining to xylose, because the type strain can assimilate D-xylose).

*Description*. In YM broth after 3 days at 25°C, cells are ovoid to elongate (2–5.5 × 3.5–7 μm) and occur singly or in pairs (Figure 3B). Budding is multilateral. In YM broth after a month, a sediment is formed, but a pellicle is not observed. On YM agar after 3 days at 25°C, colonies are white to cream-colored, buttery, convex, and smooth, with entire margins (Figure 3A). After 7 days at 25°C on a Dalmau plate culture with CM agar, hyphae are not produced, but pseudohyphae are present (Figure 3C). Asci or signs of conjugation were not observed on sporulation media.

Glucose, galactose, trehalose and D-xylose (weak) are fermented. Glucose, galactose, L-sorbose, D-glucosamine, D-xylose, L-arabinose, L-rhamnose, sucrose, maltose, trehalose, methyl α-D-glucoside, cellobiose, salicin, arbutin, lactose, melezitose, ribitol, xylitol, D-glucitol, D-mannitol, galactitol, D-glucono-1, 5-lactone, D-gluconate, succinate, and citrate are assimilated. No growth was observed in D-ribose, D-arabinose, melibiose, raffinose, inulin, glycerol, erythritol, *myo*-inositol, 2-Keto-D-gluconate, D-glucuronate, DL-lactate, methanol or ethanol. In nitrogen-assimilation tests, growth is present on ethylamine, L-lysine, glucosamine, and D-tryptophan, while growth is absent on nitrate, nitrite, cadaverine, creatine, creatinine, and imidazole. Growth is observed at 35°C but not at 37°C. Growth in vitamin-free medium is present, but growth in the presence of 0.01% cycloheximide, 10% NaCl with 5% glucose and 1% acetic acid is absent. Starch-like compounds are not produced. Urease activity and diazonium blue B reactions are negative. The G + C content of the genome of the strain NYNU 174173^T^ is 50.87 mol%, its approximate size is 15.3 Mbp.

*Note*. Members of *Cl. xylosa* have intraspecific variability in phenotypic characteristics. Strain NYNU 174173^T^ is able to assimilate maltose, salicin and ribitol, while strain NYNU 168193 do not.

*Clavispora paralusitaniae* F.L. Hui & C.Y. Chai sp. nov. (Figure 4).

*Mycobank No*.: 845628.

*Type*. China, Henan Province, Funiu Mountain Nature Reserve, rotting wood, 2 August, 2016, NYNU 161120 (holotype CICC 33276, culture ex-type CBS 15234).

*Etymology*. *Clavispora paralusitaniae* (pa.ra.lu.si.ta.ni’ae. N.L. gen. n. *paralusitaniae*, similar to *lusitaniae*, referring to the close relationship to *Clavispora lusitaniae*).

*Description*. In YM broth after 3 days at 25°C, cells are ovoid to elliptical (2–7 × 2–8 μm) and occur singly or in pairs (Figure 4B). Budding is multilateral. In YM broth after a month, a sediment is formed, but a pellicle is not observed. On YM agar after 3 days at 25°C, colonies are white to cream-colored, buttery, raised, and smooth, with entire margins (Figure 4A). After 7 days at 25°C on a Dalmau plate culture with CM agar, hyphae are not produced, but pseudohyphae are present (Figure 4C). Asci or signs of conjugation were not observed on sporulation media.

Glucose, galactose, maltose (weak), sucrose, trehalose (weak), and cellobiose are fermented. Glucose, galactose, l-sorbose, D-glucosamine, D-xylose, L-rhamnose, sucrose, maltose, trehalose, methyl α-D-glucoside, cellobiose, salicin, arbutin, melezitose, inulin (weak), glycerol, ribitol, xylitol, D-glucitol, D-mannitol, D-glucono-1, 5-lactone, 2-keto-D-gluconate (weak), D-gluconate, DL-lactate, succinate, and citrate are assimilated. No growth was observed in D-ribose, L-arabinose, D-arabinose, melibiose, lactose, raffinose, erythritol, galactitol, *myo*-inositol, D-glucuronate, methanol or ethanol. In nitrogen-assimilation tests, growth is present on ethylamine, L-lysine, cadaverine, glucosamine, and D-tryptophan, while growth is absent on nitrate, nitrite, creatine, creatinine, and imidazole. Growth is observed at 37°C but not at 40°C. Growth in vitamin-free medium is present, but growth in the presence of 0.01% cycloheximide, 10% NaCl with 5% glucose and 1% acetic acid is absent. Starch-like compounds are not produced. Urease activity and diazonium blue B reactions are negative. The G + C content of the genome of the strain NYNU 161120^T^ is 44.51 mol%, its approximate size is 12.6 Mbp.

*Note*. Strains of *Cl. paralusitaniae* exhibits minor differences in phenotypic characteristics. Strain NYNU 168424 assimilates D-arabinose but strains NYNU 161120^T^ and NYNU 167235 do not. Additionally, fermentation of galactose is positive for two strain NYNU 161120^T^ and NYNU 167235, while that of strain NYNU 168424 is negative.

## Discussion

In this study, we isolated five novel yeast strains from rotting wood samples collected in Henan Province, central China. Phylogenetic analyses based on a single-locus (LSU D1/D2) and a two-locus (ITS and LUS D1/D2) approach showed that the five strains formed two robust groups, represented by strains NYNU 174173^T^ and NYNU 161120^T^, together with their relatives (*C. thailandica* CBS 10610^T^ and *Cl. lusitaniae* CBS 6936^T^), and were obviously separate from other type strains in the *Clavispora* clade. Pairwise sequence comparison of the LSU D1/D2 domains and the ITS regions of the two novel strains with related species showed that they had lower similarity values than the common threshold for species differentiation in yeast (Kurtzman and Robnett, 1998; Scorzetti et al., 2002). In addition, they shared similar physiological and biochemical characteristics with species in the *Clavispora* clade, but clearly differed from the closest known species, *C. thailandica* CBS 10610^T^ and *Cl. lusitaniae* CBS 6936^T^ (Table 3). Thus, the two isolates represented two novel species in the genus *Clavispora*. This distinction was supported by genomic analyses. Based on genomic G + C content, strains NYNU 174173^T^ and NYNU 161120^T^ were clearly distinct from *C. thailandica* CBS 10610^T^ and *Cl. lusitaniae* CBS 6936^T^, respectively (Table 4). When strains NYNU 174173^T^ and NYNU 161120^T^ were compared with each other or with their closest relatives, the genomic relatedness with ANI and DDH values was lower than the proposed threshold value for species delineation (Table 5; Lachance and Lee, 2020; Cadež et al., 2021). These results, together with the robust phylogenetic positions and the differences in phenotypic characteristics, indicated that the two isolated strains represented two novel species in the genus *Clavispora*. We therefore propose two new species, namely *Clavispora xylosa* sp. nov. and *Clavispora paralusitaniae* sp. nov., to accommodate these yeasts.

## Data availability statement

The datasets presented in this study can be found in online repositories. The names of the repository/repositories and accession number(s) can be found in the article/Supplementary material.

## Author contributions

C-YC and YL isolated the strains and performed the taxonomic characterization and genomic analyses. C-YC prepared the draft manuscript, and the tables and figures. Z-LY and F-LH designed the study and revised the manuscript. All authors contributed to the article and approved the submitted version.

## Funding

This research was funded by the National Natural Science Foundation of China (Project no. 31570021) and the State Key Laboratory of Motor Vehicle Biofuel Technology, Henan Tianguan Enterprise Group Co., Ltd., China (no. 2018001).

## Conflict of interest

Z-LY was employed by the company Henan Tianguan Enterprise Group Co., Ltd.

The remaining authors declare that the research was conducted in the absence of any commercial or financial relationships that could be construed as a potential conflict of interest.

## Publisher’s note

All claims expressed in this article are solely those of the authors and do not necessarily represent those of their affiliated organizations, or those of the publisher, the editors and the reviewers. Any product that may be evaluated in this article, or claim that may be made by its manufacturer, is not guaranteed or endorsed by the publisher.

## Footnotes

^1^ https://www.ezbiocloud.net/tools/ani

^2^ http://ggdc.dsmz.de/ggdc.php
