# Corrigendum: Phylogenetic and genomic analyses of two new species of *Clavispora* (*Metschnikowiaceae, Saccharomycetales*) from Central China

**DOI:** 10.3389/fmicb.2022.1093453

**Published:** 2022-12-05

**Authors:** Chun-Yue Chai, Ying Li, Zhen-Li Yan, Feng-Li Hui

**Affiliations:** ^1^College of Life Science and Agricultural Engineering, Nanyang Normal University, Nanyang, China; ^2^Research Center of Henan Provincial Agricultural Biomass Resource Engineering and Technology, Nanyang Normal University, Nanyang, China; ^3^State Key Laboratory of Motor Vehicle Biofuel Technology, Henan Tianguan Enterprise Group Co., Ltd, Nanyang, China

**Keywords:** *Clavispora paralusitaniae* sp. nov., taxonomy, rotted wood-inhabiting yeast, genomic analyses, phylogeny, *Clavispora xylosa* sp. nov.

In the published article Figures 4A–C were not cited in the article. The citations have now been inserted in **Materials and methods**, section “*Phenotypic characterization*,” paragraph one. The paragraph should be corrected as follows:

“Phenotypic characterization was carried out for strains NYNU 174173^T^ and NYNU 161120^T^ using standard methods (Kurtzman et al., 2011). The yeasts shared similar phenotypic characteristics with other species in the *Clavispora* clade. Colonies were white to cream-colored, buttery, convex, and had an entire margin (Figures 3A, 4A). Cells were ovoid to elongate, proliferated by multilateral budding (Figures 3B, 4B), and formed pseudohyphae but not hyphae (Figures 3C, 4C). They were fermentative and could not assimilate nitrate as a nitrogen source. Their growth in vitamin-free medium was inconsistent with previous descriptions of the *Clavispora* clade (Jindamorakot et al., 2007; Yurkov et al., 2009; Lachance and Phaff, 2011), but studies of other closely related species demonstrated that this trait must be considered variable in this clade. Neither conjugation nor ascospores were observed in single or mixed cultures on sporulation media, suggesting that these strains represent anamorphs of the genus *Clavispora*.”

In the published article, there was an error in **Materials and methods**, Table 1 page 3. At the first column and the fourth row, the word “*paralusitanie*” was redundant and should be deleted, the strain should be “*Clavispora paralusitaniae*.” The corrected Table 1 and its caption appear below.

**Table 1:** Novel yeast strains isolated from rotting wood.

**Strain**	**Source**	**Location**
*Clavispora xylosa*		
NYNU 174173^T^	Rotting wood	Baotianman Nature Reserve, Henan, China
NYNU 168193	Rotting wood	Funiu Mountain Nature Reserve, Henan, China
*Clavispora paralusitaniae*		
NYNU 167235	Rotting wood	Funiu Mountain Nature Reserve, Henan, China
NYNU 168424	Rotting wood	Baotianman Nature Reserve, Henan, China
NYNU 161120^T^	Rotting wood	Funiu Mountain Nature Reserve, Henan, China

In the published article, there were two errors. The collection date of the strains are incorrect.

A correction has been made to **Results**, section “*Taxonomy*,” paragraph 3. The sentence should be corrected as follows:

“*Type*. China, Henan Province, Baotianman Nature Reserve, rotting wood, 3 August, 2016, NYNU 174173 (holotype CICC 33277, culture ex-type CBS 15236).”

A correction has been made to **Results**, section “*Taxonomy*,” paragraph 10. The sentence should be corrected as follows:

“*Type*. China, Henan Province, Funiu Mountain Nature Reserve, rotting wood, 2 August, 2016, NYNU 161120 (holotype CICC 33276, culture ex-type CBS 15234).”

The authors apologize for these errors and state that these do not change the scientific conclusions of the article in any way. The original article has been updated.

## Publisher's note

All claims expressed in this article are solely those of the authors and do not necessarily represent those of their affiliated organizations, or those of the publisher, the editors and the reviewers. Any product that may be evaluated in this article, or claim that may be made by its manufacturer, is not guaranteed or endorsed by the publisher.
